# The Analysis of Acute and Subacute Toxicity of Silver Selenide Nanoparticles Encapsulated in Arabinogalactan Polymer Matrix

**DOI:** 10.3390/polym14153200

**Published:** 2022-08-05

**Authors:** Evgeniy A. Titov, Larisa M. Sosedova, Mikhail A. Novikov, Marina V. Zvereva, Viktor S. Rukavishnikov, Oleg L. Lakhman

**Affiliations:** 1 East Siberian Institute of Medical and Ecological Research, 665827 Angarsk, Russia; 2 A.E. Favorsky Irkutsk Institute of Chemistry, 664033 Irkutsk, Russia

**Keywords:** nanocomposite, laboratory animals, silver selenide, toxicity, morphology, brain, liver, kidney

## Abstract

The acute and subacute toxicity of a newly synthesized silver selenide nanoparticles encapsulated in a natural polymeric matrix of arabinogalactan study has been studied. The nanocomposite is a promising material for the design of diagnostic and therapeutic drugs. It can also be used for the preparation of fluorescent labels and in thermal oncotherapy. The employment of binary nanocomposites enables one to unveil the potential hidden in metals which constitute these composites. The study of acute toxicity, carried out by the oral administration of nanocomposites at a dose of 2000 mg/kg, has shown that the compound belongs to low-toxic substances of the 5th hazard class. With the subacute oral administration of nanocomposites at a dose of 500 μg/kg, slight changes are observed in the brain tissue and liver of experimental animals, indicating the development of compensatory–adaptive reactions. In the kidneys, the area of the Shumlyansky–Bowman chamber decreases by 40.5% relative to the control group. It is shown that the application of the protective properties of selenium, which is contained in the composite, helps to reduce the toxicity of silver.

## 1. Introduction

Silver selenide is a promising material for the creation of biomedical theranostic preparations. Due to the fact that the silver selenide luminesce is in the region of electromagnetic radiation that is not absorbed by biological tissues, the application of its quantum dots (QDs) as fluorescent labels seems to be prospective [1,2]. Silver selenide can be employed in oncology to improve the efficiency of photothermal therapy, the process of destroying cancer cells using infrared radiation as a heat source [1,3,4]. Silver nanoparticles are among the strongest natural antiseptics and have pronounced bacteriostatic and bactericidal effects [5,6]. They are also capable of overcoming the blood–brain barrier and accumulating in the brain tissue [7,8]. At the same time, silver nanoparticles are known to exhibit neurotoxic [7,8,9] and hepatotoxic [10] action at high doses. In turn, selenium, in addition to antibacterial properties [11], has cytostatic effects [12] and can suppress tumor growth [12,13,14]. The use of binary nanocomposites (which contain both Ag and Se) enables the unveiling of the potential hidden in the metals which constitute these composites. All of the above effects make the silver selenide nanocomposite a promising material for theranostics.

**Figure 1:**
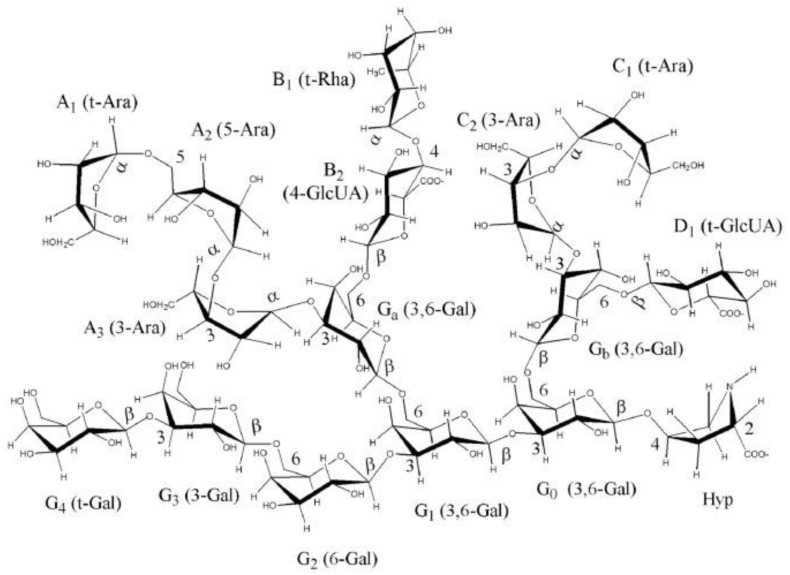
Fragment of AG macromolecule [18].

The improvement of the bioavailability of metal nanoparticles as well as the reduction of their possible toxicity represents an urgent challenge. In this regard, the synthesis of metal-containing nanocomposites using high-molecular compounds, for example, arabinogalactan (AG) of Siberian larch [15,16], appears to be a promising direction. The natural polymer AG is a water-soluble white or cream-colored powder without taste and odor, the production technology of which was patented [17]. The AG macromolecule (Figure 1) is represented by the residues of two monosaccharides: galactose and arabinose. The structure of the polysaccharide has a main chain consisting of galactopyranosyl units connected by β-bonds (1 → 3) and a side chain representing various combinations of galactopyranosyl and arabinofuranosyl residues connected by β-bonds (1 → 6) [18].

The polymer is isolated from larch wood, where its content reaches 15%. The specified polymer is a biologically active substance with a wide spectrum of activity. It is found that it possesses good gastroprotective, immunomodulatory, membranotropic and antioxidant properties [19,20,21]. It is shown that AG significantly weakens the effect of chemical toxicants on oxidation processes involving free radicals [22]. In addition, earlier AG already successfully demonstrated itself as an effective stabilizer of a number of different nanoparticles (noble metals, metal oxides, elemental chalcogenes, etc.). The obtained nanocomposites combine good water solubility, high aggregative and kinetic stability, high biocompatibility as well as a number of specific physical–chemical (optical, luminescent and magnetic) and biological (antimicrobial, antioxidant, adaptogenic, immunomodulatory and antianemic) properties due to the presence of nano-sized inorganic components. Thus, the availability of arabinogalactan, its excellent water-solubility and the expressed stabilizing properties (the z-potential of some composites is up to −70 eV) with a rather narrow molecular weight distribution Mw (42.3–45.2 kDa) allow for the water-soluble stable nanomaterials based on it to be obtained, which combine all the above features of both arabinogalactan and an inorganic nanophase [23,24,25].

The unique characteristics of silver selenide nanoparticles encapsulated in a natural polymer matrix of AG open up wide possibilities for its application, at the same time being possible reasons for adverse effects.

In the present work, the morphofunctional state of the tissue of the sensorimotor zone of the cerebral cortex and the hepato-renal system during the acute and subacute administration of silver selenide nanoparticles encapsulated in a natural AG polymer matrix has been evaluated.

## 2. Materials and Methods

### 2.1. Chemical Synthesis of a Water-Soluble Ag_2_Se-Containing Nanocomposite Based on AG

The nanocomposite was synthesized according to the slightly modified method described in [2]. Namely, an aqueous solution containing 0.178 g AgNO_3_ was added to 100 mL of aqueous solution containing 3.4 g of AG under vigorous stirring at room temperature. After 10 min of stirring, 225 µL of reaction medium containing Se^2−^-anions, previously generated from powdered elemental selenium in the basic-reduction “hydrazine-hydrate-alkali” system [2], was added to the obtained transparent colorless solution (AG + AgNO_3_). The formation of Ag_2_Se nanoparticles was identified by the appearance of brown staining of the reaction medium and the absorption spectrum characteristic of Ag_2_Se. The synthesis time was 20 min. The nanocomposite was isolated by precipitation from the reaction medium in a 5-fold excess of ethanol.

The optical absorption spectrum of the nanocomposite aqueous solution was recorded on a Perkin Elmer Lambda 35 spectrophotometer (Waltham, MA, USA) in a 1 cm quartz cuvette. X-ray diffractometer Bruker D8 ADVANCE (Waltham, MA, USA), (Cu radiation, Goebel mirror) was used for the X-ray diffraction study. Transmission electron microscopy (TEM) was performed on a Leo 906 E transmission electron microscope (Zeiss, Jena, Germany). The size distribution of nanoparticles was determined by statistical processing of microphotographs. The elemental composition was determined by X-ray energy dispersive microanalysis on an electron scanning microscope (SEM) Hitachi TM 3000 with X-ray detector SDD XFlash 430-4 (Tokyo, Japan)and on CHNS-analyzer Flash 2000 company Thermo Scientific (Waltham, MA, USA). AG—Found (%): C, 39.5; H, 6.39; Ag_2_Se-AG—Found (%): C, 38.5; H, 5.6; Ag, 2.9%; Se, 1.1%.

### 2.2. Animals and Experimental Design

For the study purposes, animals were randomly selected, labeled with individual identifiers, and kept in their cages for three to four individuals each for 5 days prior to dosing to allow them to adapt in the laboratory. The absence of external signs of diseases and the homogeneity of groups by body weight (±20%) were considered as a criterion for the acceptability of randomization. Prior to dosing, the animals remained fasted for 3–4 h with free access to water. Animals of the experimental and control groups were kept under the same environmental conditions in the chambers.

Experimental animals were born by their own reproduction in the vivarium of Federal State Budgetary Scientific Institution “East Siberian Institute of Medical and Ecological Research” (FSBSI ESIMER) and kept on a standard diet. Maintenance and care of experimental animals were carried out in accordance with the interstate standard GOST 33216-2014 (Russia).

All animal experiments were approved by the ethical committee of FSBSI East-Siberian Institute of Medical and Ecological Research (identification code: E06/21; date of approval: 24 June 2021, amended/approvals every 6 months). All manipulations with experimental animals were performed in accordance with the rules of humane treatment of animals in accordance with the requirements of the International Recommendations for Biomedical Research Using Animals (WHO, Geneva, 1985).

### 2.3. Study of Acute Toxicity

The acute toxicity method [26] is a step-by-step procedure using a minimum number of animals of the same sex at each step. The method for determining the class of acute toxicity is based on biometric assessments with fixed doses, which are distributed over the time of administration so that it is possible to assess the substance according to the degree of danger and systematize the results in human body. Acute oral toxicity (method for determining the class of acute toxicity of the Ag_2_Se nanoparticles encapsulated in a natural polymer matrix of arabinogalactan (Ag_2_Se-AG)) was determined by the intragastric route of administration on outbred white male mice weighing 21–32 g (experimental *n* = 6, control *n* = 6).

The acute toxicity test of the Ag_2_Se-AG nanocomposite was performed using the limiting dose (2000 mg/kg of body weight). The test substance was administered orally to white mice of the experimental group with an atraumatic probe at a dose of 2000 mg/kg in 0.9% NaCl. At the same time, the volume of the injected solution did not exceed 0.5 mL. Dosing accuracy was achieved by changing the volume of the injected solution at its constant concentration. Animals of the control group were orally administered in an equivalent volume of 0.9% NaCl (placebo). The doses were prepared immediately prior to the administration. Before the introduction of the test dose, the animals were weighed and the administered dose was calculated at the rate of 2000 mg/kg of body weight. Prior to dosing, mice remained fasted for 3–4 h with free access to water. Food was absent for another 2–3 h after introduction of substance.

Animals were observed daily for 14 days, with particular attention paid to the first 4 h after administration of the test dose. The appearance and disappearance of the external signs of poisoning were assessed: changes in the skin and coat, eyes and mucous membranes, respiratory, circulatory, autonomic and central nervous systems, as well as somatomotor activity and behavior, the appearance of tremor, convulsions, salivation, diarrhea, lethargy, sleep and coma [27]. Then the mice were sacrificed by the method of dislocation of the cervical vertebrae and macroscopic analysis of the internal organs was performed.

### 2.4. Study of Subacute Toxicity

The study of the subacute effect of the nanocomposite was carried out with intragastric administration of Ag_2_Se-AG on white male rats weighing 200–220 g (experimental *n* = 10, control *n* = 10). In case of subacute exposure, the experimental group of animals was intragastrically injected with the studied nanocomposite at a dose of 500 mg/kg of body weight in 1 mL of distilled water for 10 days. This dose was chosen based on the results of previous investigations and was 1/4 of LD_50_. The choice of dose is due to previous studies of the toxic properties of nanocomposites of other metals (Ag, Bi, Fe, etc.) [28,29,30,31,32,33], indicating the development of clear and persistent signs of pathology with the introduction of this dose. Control animals (*n* = 10) received 1 mL of distilled water in the same mode. For histological studies of the nervous tissue, the animals were euthanized by decapitation.

### 2.5. Histological Investigation

The brain, liver and kidneys were isolated from each animal under study and fixed in a neutral buffered 10% formalin solution (BioVitrum, St. Petersburg, Russia). The brain was dehydrated with isopraponol (BioVitrum, St. Petersburg, Russia) and placed in HistoMix homogenized paraffin medium for histological studies (BioVitrum, Russia). Using a HM 400 microtome (Microm, Munchen, Germany), serial frontal paraffin sections of the brain were made for subsequent staining with hematoxylin–eosin for survey microscopy. The nervous tissue of the temporo-parietal zone of the sensorimotor cortex of the brain was studied as a nerve center that provides the regulation of the basic physiological functions of the body and complex forms of behavior [9]. The liver and kidneys after fixation were examined by alcohols of increasing concentrations and embedded in paraffin. Section 3, Section 4 and Section 5 microns thick were prepared from paraffin blocks, which were stained with hematoxylin and eosin according to the generally accepted method [34]. In the brain preparations, the number of neurons per unit area, astroglial cells and dead neurons were counted, and the number of neuronophagy events were counted using the ImageScope M program (Russia). The number of Kupffer stellate macrophages and the number of polynuclear hepatocytes were counted in the liver tissue. The area of the Shumlyansky–Bowman chamber was evaluated in the kidney tissue. The obtained sections were examined using an Olympus BX 51 light-optical research microscope (Tokyo, Japan) with input of microimages into a computer using an Olympus E420 camera (Tokyo, Japan).

### 2.6. Statistical Analyses

Statistical analysis of the research results was carried out using the Statistica 6.1 software package. To compare the groups, the Mann–Whitney U-test was used. Changes in the studied parameters were considered statistically significant at a significance level of *p* ≤ 0.05.

## 3. Results

### 3.1. Characteristics of the Synthesized Nanocomposite Ag_2_Se-AG

The water-soluble Ag_2_Se-containing nanocomposite based on AG with a quantitative content of the inorganic phase of 4%w was synthesized out in aqueous medium via the ion-exchange interaction of selenide anions Se^2−^ (generated from bulk powder samples of elemental selenium in a basic reduction system “hydrazine hydrate—alkali” (Equation (1)) and Ag^+^ ions, according to Equation (2), in the presence of AG macromolecules.

2 Se + 4 KOH + N_2_H_4_ × H_2_O = 2 K_2_Se + 5 H_2_O + N_2_↑ (1)

2AgNO_3_ + K_2_Se → Ag_2_Se↓ + 2KNO_3_ (2)

The passivation of the energy-saturated surface of Ag_2_Se nanoparticles and the support of their aggregative stability are probably performed by the adsorption of the polysaccharide macromolecules on their surface (steric stabilization) as well as due to the electrostatic stabilization of the Ag_2_Se particle surface by the highly-polar functional AG groups (hydroxyl, terminal carbonyl). A single hybrid stable water-soluble system “nanocore—Ag_2_Se/shell—polysaccharide matrix” is formed.

**Figure 2:**
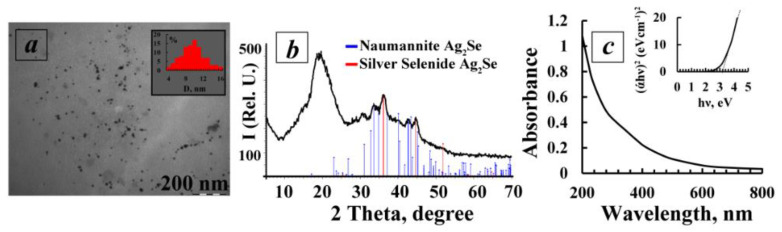
(**a**)—Microphotograph and particle size distribution (inset) of Ag_2_Se nanoparticles in AG polysaccharide matrix; (**b**)—diffractogram of Ag_2_Se-AG nanocomposite (4%w Ag_2_Se); (**c**)—absorption spectrum of 0.1% of Ag_2_Se-AG nanocomposite (4%w Ag_2_Se) water solution and its Tauc plot of (αhν)^2^ vs. (hν) (inset).

According to the data of transmission electron microscopy, the Ag_2_Se-AG nanocomposite (4%w Ag_2_Se) is formed as spherical Ag_2_Se nanoparticles dispersed in the polysaccharide matrix of AG. The particle size varies between 4–16 nm with an average value of 9.6 nm. (Figure 2a). Using XRD, it was found that the obtained Ag_2_Se-containing nanocomposite has a two-phase amorphous–crystalline structure. Its diffractogram is presented by a halo in the region of 10–24°, corresponding to the amorphous AG phase, and also by a set of reflexes of different intensities in the region of 33.6°, 36.1° and 45.1° (JCPDS Card No. 24-1041), characterizing the presence of silver selenide with a cubic crystal lattice (α-Ag_2_Se) in the composite obtained [35]. In addition, the diffractogram shows a set of low-intensity reflexes in the region of 31–70°, corresponding to orthorhombic crystal lattice β-Ag_2_Se (on the type of mineral Naumannite) [36] (Figure 2b).

The average size of Ag_2_Se nanocrystallites, calculated by the Scherrer formula, is 11.2 nm which correlates well with the TEM data (Figure 2c). The experimentally obtained value of cell parameter a (0.4962) agrees well with that of the reference sample of cubic silver selenide (a = 0.4983 nm).

The optical properties of an aqueous solution of Ag_2_Se nanocomposite (4%w Ag_2_Se) were studied by optical spectroscopy in the visible region of the spectrum at room temperature. It was found that the absorption spectrum is characterized by the absence of well-resolved maxima, probably due to the relatively large size of Ag_2_Se nanoparticles and their wide dispersed distribution, which is confirmed by TEM data.

The value of the optical band gap energy (*Eg*) of the Ag_2_Se nanoparticles in the AG matrix can be calculated by Tauc’s plot method. The basis of this method is the suggestion of the possibility to present the absorption coefficient *α* in the form of the equation:

(*α* × *hν*)^1/*γ*^ = *B*(*hν* − *Eg*) (3)

where *Eg* is the band gap energy, *h* is Planck’s constant, *ν* is the photon frequency and *B* is a constant. We chose the factor *γ*, which depends on the nature of the electron transition, as 1/2, assuming the direct character of the transitions [37]. According to the data obtained, the optical gap energy of the synthesized Ag_2_Se nanoparticles was higher (3.2 eV) than the value of 0.16 eV reported earlier for bulk Ag_2_Se [38]. Presumably, the increase of the gap in Ag_2_Se nanoparticles compared to the value of bulk silver selenide may be due to the decrease of the particle size and appearance of the quantum confinement effect.

### 3.2. Acute Toxicity Study

The measurement of body weight of laboratory animals is an integral indicator of the state of the organism. During the initial weighing of mice before the introduction of the test nanocomposite, the individuals of the studied groups almost did not differ from each other in weight, being varied within 30–35 g. When weighed one week and 2 weeks after administration, the weight of the animals either increased or remained at the same level. Thus, oral administration of the nanocomposite during observation for 14 days did not decrease the body weight in any case, *p* ≥ 0.05 (Table 1).

During the entire observation, there was no mortality of animals from the experimental group. As in the latter, the observation of mice from the control group also did not reveal changes in behavior, condition of wool or in the consumption of water and food.

A macroscopic investigation of the internal organs of mice euthanized after 14 days of observation showed no differences between the experimental and control groups. Mice had the correct constitution and obtained satisfactory nutrition. The coat had a neat appearance and no foci of baldness were determined. Visible mucous membranes were shiny, smooth and pale in color. Thoracic and abdominal cavities did not contain effusion. The position of the internal organs did not have any disorders. The thyroid gland was of normal size and shape and reddish in color.

The size and shape of the heart did not change. The heart muscle was moderately dense and brownish in color. The lumen of the trachea and large bronchi were uniformly wide. The lungs were easily collapsed when the chest was opened and the surface was of a uniform pale pink color. The tissue of the lungs was airy to the touch.

The stomach had the usual dimensions and its lumen was filled with food contents. The mucous membrane was folded, homogeneous and pinkish in color. Irritation and hyperemia was not observed. The shape and size of the liver did not change. The liver tissue was moderately plethoric. The pancreas was pale pink. The size and shape of the spleen corresponded to those of the control mice. The tissue of the spleen had a moderately dense consistency and dark cherry color. Kidneys were of normal color with a clearly visible cortical immedulla. The membranes of the brain were shiny, thin and smooth. No ventricular expansion was observed on the frontal sections.

It should be noted that a macroscopic investigation of the internal organs of animals from the control group also did not show any pathological changes.

Based on the results obtained, due to the absence of mortality associated with the studied nanocomposite in animals dosed at one stage, the further research seems to be unreasonable [39].

Thus, according to the data of a macroscopic study, acute oral administration of the Ag_2_Se-AG nanocomposite at the maximum dose to white male mice did not cause visible changes in the internal organs, brain and tracheal and stomach mucosa. The study of acute toxicity showed that the test substance can be attributed to the 5th hazard class. Thus, this drug belongs to low-hazard substances.

### 3.3. Subacute Toxicity Study

**Figure 3:**
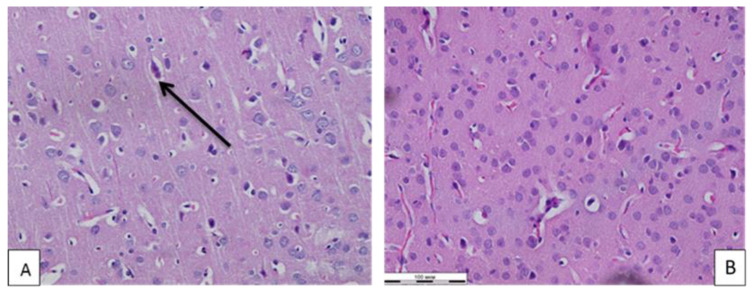
Microphoto of brain tissue of white rats of experimental (**A**) and control (**B**) groups. ↑—degeneratively altered neuron. Hematoxylin–eosin. Mag. ×400.

The study of the subacute effect of Ag_2_Se-AG on the brain tissue showed that the blood filling of the vessels of the brain substance and the state of the vascular intima were unchanged. The number of normal neurons, astroglial cells and degeneratively altered neurons (darkly stained neurons were considered degeneratively altered, without a clearly separated nucleus and cytoplasm) per unit area had no statistically significant differences from the control values. The number of neuronophagy events in animals that received the nanocomposite was statistically significantly higher than in the control group (Table 2, Figure 3).

**Figure 4:**
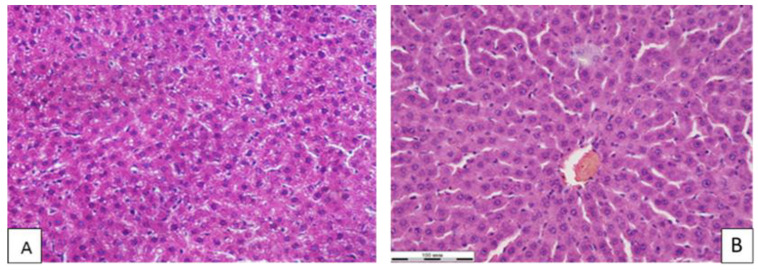
Microphoto of liver tissue of white rats of experimental (**A**) and control (**B**) groups. okr. Hematoxylin–eosin. Mag. ×400.

Thus, the exposure of the binary nanocomposites of silver selenide at a dose of 500 μg/kg had an insignificant effect on the number and structure of the population of nerve cells in the sensorimotor cortex of albino rats. In the liver tissue, the blood filling of the sinusoidal capillaries, central veins and veins of the portal tracts was normal. Portal tracts were not dilated and were without signs of sclerosis and inflammation. The beam-radial structure of the hepatic lobules was preserved. The number of stellate Kupffer macrophages in the sinusoidal capillaries did not differ from the control group. At the same time, the number of polynuclear hepatocytes was statistically significantly higher than in the control group (Table 2, Figure 4).

**Figure 5:**
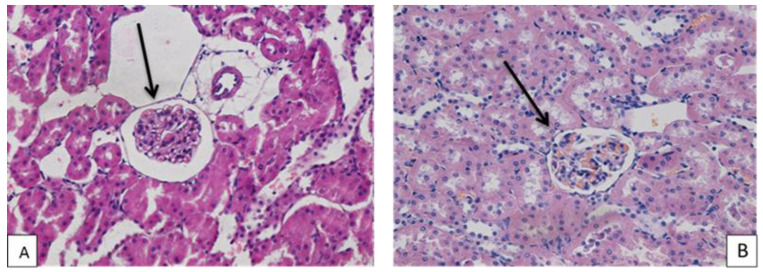
Microphoto of kidney tissue of white rats of experimental (**A**) and control (**B**) groups. ↑—Shumlyansky–Bowman chamber. Hematoxylin–eosin. Mag. ×400.

In the kidney tissue, the blood filling of the cortical and medulla of the organ was unchanged. The violations of the blood rheology in the body were not observed. The condition of the walls of the renal arteries, arterioles and interstitial space was normal. The structure of the renal glomeruli was preserved. There were no foci of inflammation or necrosis of the renal tissue. The epithelium of the distal and proximal renal tubules was intact. In the cortical substance of the kidney, a statistically significant decrease in the area of the Shumlyansky–Bowman capsule was revealed in comparison with the control group (Table 2, Figure 5).

## 4. Discussion

With the acute oral administration of the nanocomposite to white mice at a maximum dose of 2000 mg/kg of body weight, no mortality was noted during the entire observation. This, together with the absence of changes in internal organs during the macroscopic investigation, allows us to classify the studied Ag_2_Se-AG nanocomposite as a low-hazard substance (5th class of hazard).

Many researchers have shown that exposure to silver nanoparticles in low doses leads to the emergence and development of neurotoxic effects [7,8,9,11,40,41,42]. Skalska J. et al. [40] described the development of pathological changes in neuronal mitochondria (edema, decreased potential of the mitochondrial membrane) and, as a consequence, the induction of autophagy of the neurons themselves when exposed to silver nanoparticles at a dose of 0.2 mg/kg. The cytotoxic effect of low doses of silver nanoparticles on the main components of the blood–brain barrier (endothelial cells and astrocytes) was reported, as well as that directly on neurons, causing a disruption of the cell cytoskeleton and destruction of synaptic connections [7,41]. Our previous studies have shown that a silver nanocomposite encapsulated in an arabinogalactan polymer matrix, despite belonging to the IV low-hazard class of substances with LD_50_, when administered intragastrically at a dose of more than 5000 mg/kg of animal weight, disorders the structure of the nervous tissue, increases the area of mitochondria, violates the structure of nerve cells and activates the apoptosis process [43]. At the same time, silver nanoparticles encapsulated in the natural biopolymer matrix of arabinogalactan are able to penetrate through the blood–brain barrier and, having remained in the nervous tissue of the brain of rats for a long time, cause structural disturbances [43,44]. Silver nanoparticles do not reduce their cytotoxicity for nervous tissue.

In turn, exposure to individual selenium nanoparticles, also encapsulated in a polymeric matrix of arabinogalactan, at a dose of 500 μg/kg of animal body weight reduced the total number of neurons and astroglial cells per unit area in the sensorimotor zone of the cerebral cortex and increased the degeneratively altered neurons and the number of neuronophagy acts, which indirectly indicated both the penetration of the nanocomposite through the blood–brain barrier and the pronounced neurotoxic effect of selenium nanoparticles [45]. Exposure to excessive amounts of selenium can lead to the disruption of the functioning of neurotransmitter systems and the development of neurodegenerative and neuropsychiatric processes [42]. Taking into account that the toxic effect of selenium is realized by the suppression of the intercellular signals transmission [46], it can be assumed that the previously established reduction in the number of normal neurons and astroglial cells in the nervous tissue disturbs the intercellular interaction.

The absence of such effects upon exposure to silver selenide encapsulated in the arabinogalactan polymer matrix is apparently due to the simultaneous presence of nanoparticles in the nanocomposite and, possibly, to their competition for binding to the cell receptors of cerebral cortex neurons.

An increase in the number of polynuclear hepatocytes upon the subacute administration of the Ag_2_Se-AG nanocomposite indicates the activation and development of compensatory repair processes in the liver and stimulation of cell regeneration mechanisms, while the constant amount of Kupffer stellate macrophages evidences no inflammatory process in the liver tissue. According to the data given in [10], silver nanoparticles at a dose of 300 µg/kg disrupt the normal blood rheology in the liver, increase the number of polynuclear hepatocytes and disorder the metabolic activity of hepatocytes. A similar effect was produced by selenium nanoparticles in an arabinogalactan matrix at a dose of 500 μg/kg [46]. The hepatotoxic effect of selenium at a low dose of 10 μg mixed with lithium was shown in studies by Pinto-Vidal, F. et al. [47]. At the same time, some studies showed the hepatoprotective effect of selenium [48,49,50]. The inconsistency of the obtained data is apparently explained by different ways, forms and doses of selenium introduction into the body of biological objects.

In the kidney tissue, the administration of Ag_2_Se-AG at a dose of 500 μg/kg showed a 40.5% decrease in the area of the Shumlyansky–Bowman capsule (a change in the area within 30% is the norm [51]), which can reduce the volume of primary urine formed, and, in turn, contribute to the difficulty of metabolic products’ excretion from the body. There were no other significant structural changes in the kidney tissue exposed to the silver selenide nanocomposite. Perhaps this is due to the nephroprotective properties of selenium [52,53,54]. Meanwhile, it is known that silver nanoparticles have a nephrotoxic effect, causing structural changes in the renal tubules and renal glomeruli [55]. Perhaps this is the reason for the slight changes in the kidney tissue when the Ag_2_Se-AG nanocomposite was administered to rats.

The administration of the nanocomposite to rats for 10 days at a dose of ¼ of LD50 had a different degree of severity depending on the place of application. Of the greatest interest was the nanocomposite effect on the tissue of the cerebral cortex. The experimental studies revealed no changes in the ratio of the cellular elements of the sensorimotor zone of the cortex. Morphological disturbances of neurons were also not observed in comparison with those in control animals. In the tissue of the cerebral cortex, attention was drawn to higher acts of neuronophagy, indicating an increase in the formation of glial nodules, through which damaged or degeneratively altered nerve cells were destroyed and removed from the body with the help of macrophages. Thus, silver selenide nanocomposites, without a pronounced neurotoxic effect, still increase the number of dead neurons. It might be possible to address this issue by studying the effects of silver selenide on brain tissue in the late post-contact period.

The conducted studies revealed that the toxicity of the silver selenide nanocomposite in the arabinogalactan polymer matrix is much less pronounced than that of silver or selenium nanoparticles alone. Apparently, this fact may be due to the competitive relationship of nanoparticles. At the same time, selenium, being a physiologically important trace element that is part of glutathione peroxidase, may be an antagonist for silver particles. The literature describes the antagonistic properties of selenium for such heavy metals as mercury, arsenic, lead and cadmium [56]. Given the great importance of selenium for the functioning of the immune, endocrine and reproductive systems, metabolism, cellular homeostasis and carcinogenesis, it can be assumed that its ability to bind and activate cell receptors is much higher than that of silver. As a result, the biological effectiveness of selenium can be more pronounced. Conversely, the biological activity of silver is suppressed. For the studied compound, it can be assumed that selenium acts as a protector, suppressing the pathological effects of silver nanoparticles and thereby protecting the cellular metabolism.

## 5. Conclusions

In conclusion, the study of the acute toxicity of a silver selenide nanocomposite has shown that the substance belongs to the low-hazard class. The evaluation of the subacute toxicity of a silver selenide nanoparticle encapsulated in an arabinogalactan polymer matrix to white rats does not reveal any significant changes in the tissue structure of the sensorimotor cortex and liver of animals, along with minor changes in the kidney tissue. In this connection, the silver selenide nanocomposite encapsulated in a polymer matrix is a promising preparation for further biomedical research.

## Figures and Tables

**Table 1:** Increase in body weight of white mice of the experimental and control groups that survived intoxication with the Ag_2_Se-AG nanocomposite.

Groups	Before Injection	One Week after Injection	Two Weeks after Injection
Experimental	32.3 ± 0.55	32.5 ± 0.56	33.5 ± 0.56
Control	31.8 ± 0.79	32.0 ± 0.77	33.0 ± 0.73

**Table 2:** Morphometric parameters of the sensorimotor zone of the cerebral cortex, liver and kidney tissues during subacute administration of the Ag_2_Se-AG nanocomposite to rats at a dose of 500 μg/kg of body weight for 10 days. Me (Q25–Q75).

Indicators	Experimental Group	Control Group	*p*
Sensorimotor Area of the Cerebral Cortex			
Number of normal neurons per unit area	117.0 (110.0–145.0)	152.0 (133.0–177.0)	0.2
Number of astroglial cells per unit area	140.0 (119.0–158.0)	156.0 (119.0–164.0)	0.85
Number of degeneratively altered neurons per unit area	8.0 (7.0–15.0)	5.0 (5.0–6.0)	0.08
Number of neuronophagy	4.0 (2.0–5.0) *	1.5 (1.0–2.0)	0.05
Liver			
Number of Kupffer stellate macrophages	167.5 (140.0–192.0)	145.0 (141.0–148.0)	0.55
Number of polynuclear hepatocytes	19.5 (19.0–26.0) *	16.0 (11.0–18.0)	0.04
Kidney			
Shumlyansky–Bowman capsule area	27,715.5 (24,260.9–31,714.8) *	32,556.5 (28,573.1–36,306.2)	0.02

Notes: * differences are statistically significant compared to the control according to the Mann–Whitney test, *p* ≤ 0.05.

## Data Availability

The data presented in this study are available from the corresponding author upon request.
